# Serum checkpoint molecules in patients with IgG4-related disease (IgG4-RD)

**DOI:** 10.1186/s13075-021-02527-6

**Published:** 2021-05-24

**Authors:** Haruki Matsumoto, Yuya Fujita, Naoki Matsuoka, Jumpei Temmoku, Makiko Yashiro-Furuya, Tomoyuki Asano, Shuzo Sato, Hiroshi Watanabe, Eiji Suzuki, Sosuke Tsuji, Shoichi Fukui, Masataka Umeda, Naoki Iwamoto, Atsushi Kawakami, Kiyoshi Migita

**Affiliations:** 1 Department of Rheumatology, Fukushima Medical University School of Medicine, 1 Hikarigaoka, Fukushima, Fukushima 960-1295 Japan; 2 Department of Rheumatology, Ohta Nishinouchi General Hospital Foundation, 2-5-20 Nishinouchi, Koriyama, Fukushima, 963-8558 Japan; 3 Department of Immunology and Rheumatology, Division of Advanced Preventive Medical Sciences, Nagasaki University Graduate School of Biomedical Sciences, Nagasaki University, Sakamoto1-7-1, Nagasaki, 852-8501 Japan

**Keywords:** IgG4-related disease, Immune checkpoint molecules, Galectin-9, T cell immunoglobulin and mucin-containing-molecule-3

## Abstract

**Background:**

Immunoglobulin G4-related disease (IgG4-RD) is characterized by increased serum IgG4 concentration and infiltration of IgG4^+^ plasma cells in the affected organs. The present study aimed to characterize the serum levels of coinhibitory checkpoint molecule, T cell immunoglobulin and mucin-containing-molecule-3 (TIM-3), and its ligand, galectin-9 (Gal-9), among IgG4-related disease in patients with IgG4-RD patients with various organ involvements.

**Methods:**

Serum samples were collected from untreated 59 patients with IgG4-RD, 13 patients with rheumatoid arthritis, and 37 healthy controls (HCs). HCs lacked chronic medical diseases or conditions and did not take prescription medications or over-the-counter medications within 7 days. Patients with IgG4-RD (n = 57) were subdivided into those with visceral involvement (n = 38) and those without visceral involvement (n = 21). Serum levels of Gal-9 and soluble TIM-3 (sTIM-3) were determined using enzyme-linked immunosorbent assay (ELISA). The results were compared with the clinical phenotypes of IgG4-RD.

**Results:**

In untreated patients with IgG4-RD, serum levels of Gal-9 and sTIM-3 were significantly higher than in RA patients as well as in healthy controls. There were significant correlations between the serum levels of Gal-9 or sTIM-3 and serum levels of IgG, BAFF, or sIL-2R. However, there was no significant correlation between the serum levels of Gal-9 or sTIM-3 and serum IgG4 concentrations. Serum levels of sTIM-3 were significantly higher in a subset of patients with visceral involvements than in those without visceral involvements. However, there was no significant difference in the serum levels of Gal-9 between IgG4-RD patients with and without visceral involvements, although both Gal-9 and sTIM-3 were elevated in untreated IgG4-RD patients, and the levels of these checkpoint molecules remained unchanged after steroid therapy.

**Conclusion:**

Serum levels of Gal-9 and sTIM-3 were significantly elevated in untreated patients with IgG4-RD. Furthermore, serum levels of sTIM-3 were significantly higher in IgG4-RD patients with visceral involvements. These checkpoint molecules could be a potentially useful biomarker for IgG4-RD and for assessing the clinical phenotypes of IgG4-RD.

## Introduction

Immunoglobulin G4-related disease (IgG4-RD) is characterized by multi-organ involvement and elevated serum IgG4 levels [1]. The most affected organs in this disease are the salivary or lacrimal glands, lymph node, pancreas, biliary tract, lung, kidney, retroperitoneum, and aorta [2, 3]. In histology, a high ratio of IgG4-positive plasma cell infiltration and fibrosis in the affected organs is a major finding in IgG4-related disease [4]. However, it is unclear whether the elevated production of IgG4 is the cause of IgG4-RD or epiphenomenon. Previous studies have shown a prominent expansion of circulating plasmablasts and a tightly restricted repertoire of plasmablasts, indicating that IgG4-RD is an antigen-driven disease [5, 6].

Galectins, a protein of the conserved lectin protein family, are important regulators of immune homeostasis. Galectins are also recognized as checkpoint molecules that regulate T cell development by the regulation of thymocyte apoptosis and can be potential regulators of bone marrow B cell development [7]. Thus far, the role of checkpoint molecules has been identified in the regulation of several immune-mediated processes in autoimmune diseases. A co-inhibitory checkpoint molecule, Galectin-9 (Gal-9), plays a critical role in T cell and B cell regulation, wherein the interaction between T cell immunoglobulin and mucin-containing-molecule-3 (TIM-3) and its ligand, Gal-9, is involved in the regulation of immune responses and autoimmunity [8, 9]. We previously reported the association between the Gal-9/TIM3 pathway and rheumatic disease such as rheumatoid arthritis (RA), systemic lupus erythematosus, and adult-onset Still’s disease [10–13]. From the point of view of immune checkpoint molecules, IgG4-related pleural disease occurred as an immune-related adverse event of programmed cell death-ligand 1 inhibitor [14]. The relation between the Gal-9/TIM-3 pathway and IgG4-RD is not clear, but various immune checkpoint molecules may be involved in the pathogenesis of IgG4-RD. Furthermore, it has been shown that T follicular helper (Tfh) cells are important for the germinal center formation and IgG4 class switching and can be implicated in the immune-pathophysiology of IgG4-RD [15]. Considering that the checkpoint molecules expressed on Tfh cells are involved in B cell selection and class switching [16], we hypothesized that the dysregulated checkpoint molecules participate in the pathogenesis of IgG4-RD. In the present study, we evaluated the circulating co-inhibitory molecules, Gal-9 and soluble TIM-3 (sTIM-3), in patients with IgG4-RD.

## Methods

### Patients and study design

A total of 59 patients with IgG4-RD were included in this study. All IgG4-RD patients were diagnosed at the Department of Rheumatology of Fukushima Medical University and the Department of Immunology and Rheumatology of Nagasaki University between February 2007 and November 2020. The clinico-demographic data were retrospectively collected from Medical Records. In those 59 patients, post-treatment data were collected from patients. As controls, 37 healthy controls (HCs) were included. HCs lacked chronic medical diseases or conditions and did not take prescription medications or over-the-counter medications within 7 days. An additional independent set of 13 patients with untreated rheumatoid arthritis (RA) was included to compare the value of Gal-9 and sTIM-3. Among the 13 patients of RA, 7 (53.8%) were female, and their median age was 65 years (17–88). The serum samples of these patients were properly stored at −20 °C.

This study was conducted in accordance with the principles of the Declaration of Helsinki. Ethical approval for this study (No.29317) was provided by the Ethics Committee of Fukushima Medical University.

### Examination of biochemical markers and imaging

Laboratory study included white blood cell count (WBC), hemoglobin (Hb), platelets (PLT), serum immunoglobulin G (IgG), serum immunoglobulin G4 (IgG4), the ratio of IgG4/IgG, C-reactive protein (CRP), compliment 3 (C3), compliment 4 (C4), soluble interleukin 2 receptor (sIL-2R), and lactate dehydrogenase (LDH). All patients underwent contrast-enhanced computed tomography (CT) or plain CT scan, and some patients were evaluated by ultrasound, radiography, magnetic resonance imaging, and positron emission CT.

### Classification of IgG4-RD

We diagnosed IgG4-RD based on the 2019 ACR/EULAR classification criteria for IgG4-RD. The 2019 ACR/EULAR classification criteria for IgG4-RD include clinical, serological, radiological, and pathological findings. A patient is classified if a cumulative score of 20 or more points is obtained [17].

Furthermore, IgG4-RD patients were divided into two groups depending on the presence or absence of visceral involvement. Visceral involvement is defined as the complication of the lung pancreas, bile duct, kidney, retroperitoneal fibrosis, aorta, and prostate.

### ELISA methods

The serum concentrations of Gal-9, sTIM-3, and B cell-activating factor of the tumor necrosis factor family (BAFF) were measured using an enzyme-linked immunosorbent assay kit (R&D Systems, Minneapolis, MN, USA) according to the manufacturer’s instruction.

### Statistical analysis

The results were non-normally distributed and are presented throughout the manuscript with median and 25–75th centiles [median, IQR] and were compared by the Mann-Whitney *U* test. The comparisons between the categorical variables were analyzed by Fisher’s exact test. Correlations between continuous variables were analyzed by Spearman’s rank correlation test. Paired data were analyzed by non-parametric tests using the Wilcoxon signed-rank test. The Kruskal-Wallis test was used for continuous variables for comparisons among the three groups. Post hoc pairwise analyses between the two groups were performed by the Games-Howell test. The prognostic factors for higher levels of Gal-9 and sTIM-3 were identified using a stepwise multiple logistic regression model. All data entry and statistical analyses were performed using SPSS Statistics version 22.0 (IBM, Armonk, NY). In all the analyses, a two-tailed *p* < 0.05 was considered statistically significant.

## Results

### Characteristics of IgG4-RD patients

The demographic, clinical, and laboratory characteristics of the enrolled IgG4-RD patients have been summarized in Table 1. The median age of IgG4-RD patients was 64 years (24–85 years), and the male patients were 45 (76.3%). In IgG4-RD patients, 42 patients (71%) were diagnosed as a definite diagnosis of IgG4-RD. Although 17 patients did not fulfill the 2019 ACR/EULAR classification criteria for IgG4-RD due to the lack of histopathological findings, we judged the diagnosis of IgG4-RD was clinically reasonable. Most patients had multiple organ involvement. Serum IgG4 levels were elevated in IgG4-RD patients [median 456 (IQR) 211−988 mg/dL]. Furthermore, the ratio of serum IgG4/IgG was also increased in IgG4-RD patients (median 0.240).

**Table 1:**
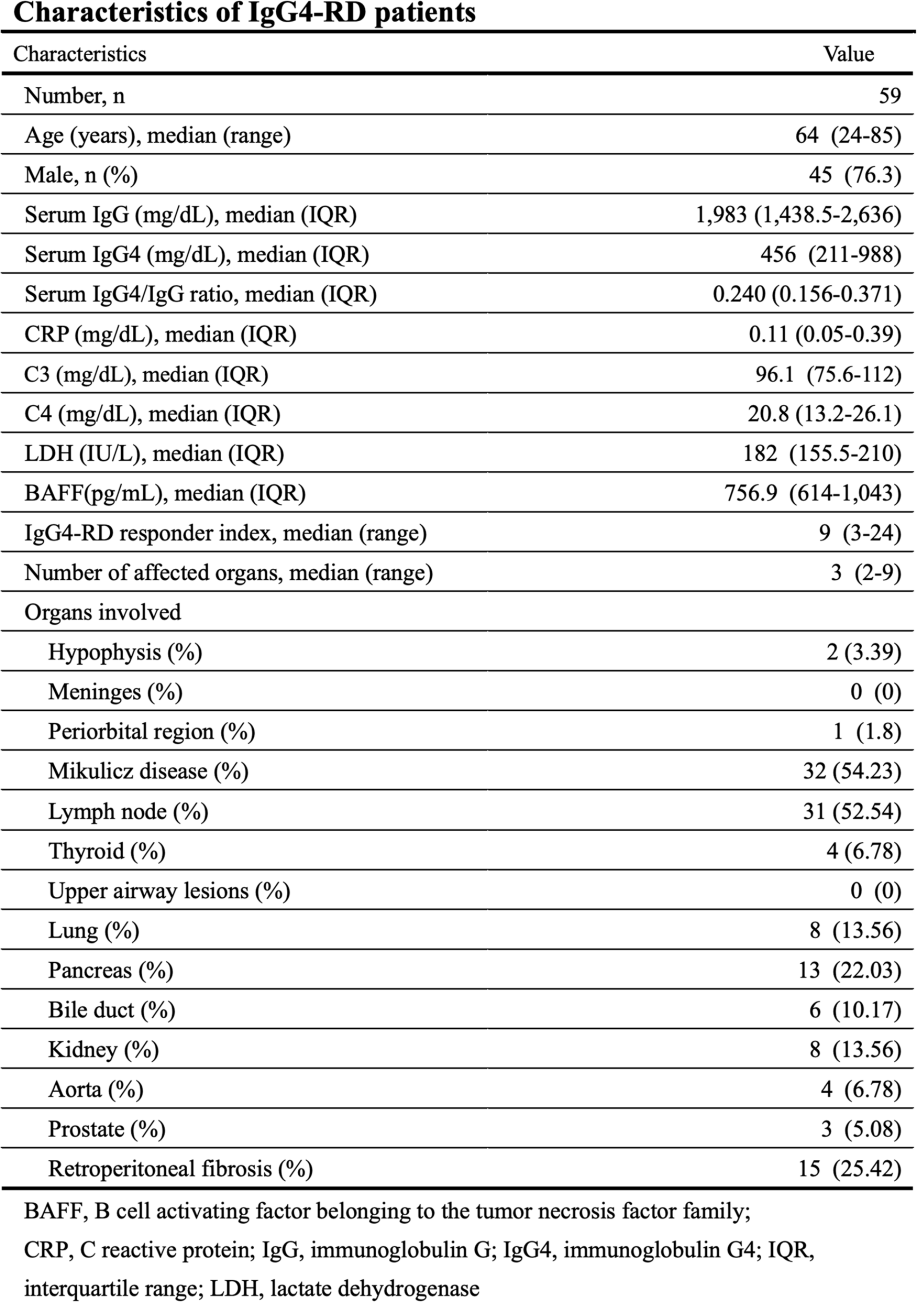
Characteristics of IgG4-RD patients

### Elevated serum levels of checkpoint molecules in IgG4-RD

**Fig. 1:**
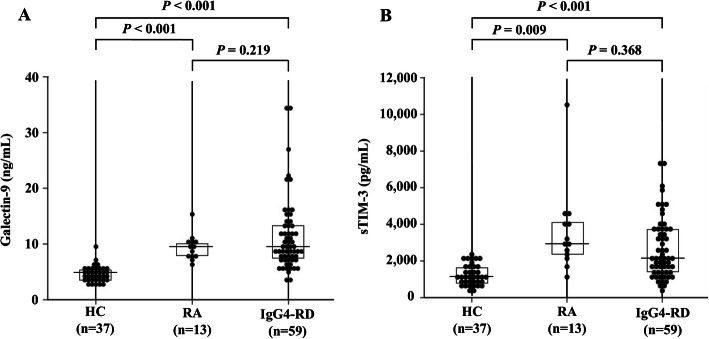
Serum levels of Gal-9 and sTIM-3 in IgG4-RD. The comparison of serum levels of Gal-9 and sTIM-3 among IgG4-RD patients (n = 59), RA patients (n = 13), and healthy controls (n = 37). **a** Serum levels of Gal-9 in IgG4-RD and RA patients were significantly higher compared to those in healthy controls. **b** Serum levels of sTIM-3 in IgG4-RD patients and RA patients were significantly higher compared to HCs. The Kruskal-Wallis test was used for continuous variables for comparisons among the three groups. Post hoc pairwise analyses between the two groups were performed by the Games-Howell test

In order to evaluate possible role in checkpoint molecules in IgG4-RD, we compared the serum levels of Gal-9. As shown in Fig. 1, the serum levels of Gal-9 were significantly higher in IgG4-RD compared to those in RA patients as well as those in HCs. Similarly, serum levels of sTIM-3 were significantly higher in IgG4-RD compared to those in HCs.

### Correlations among the biomarkers

**Fig. 2:**
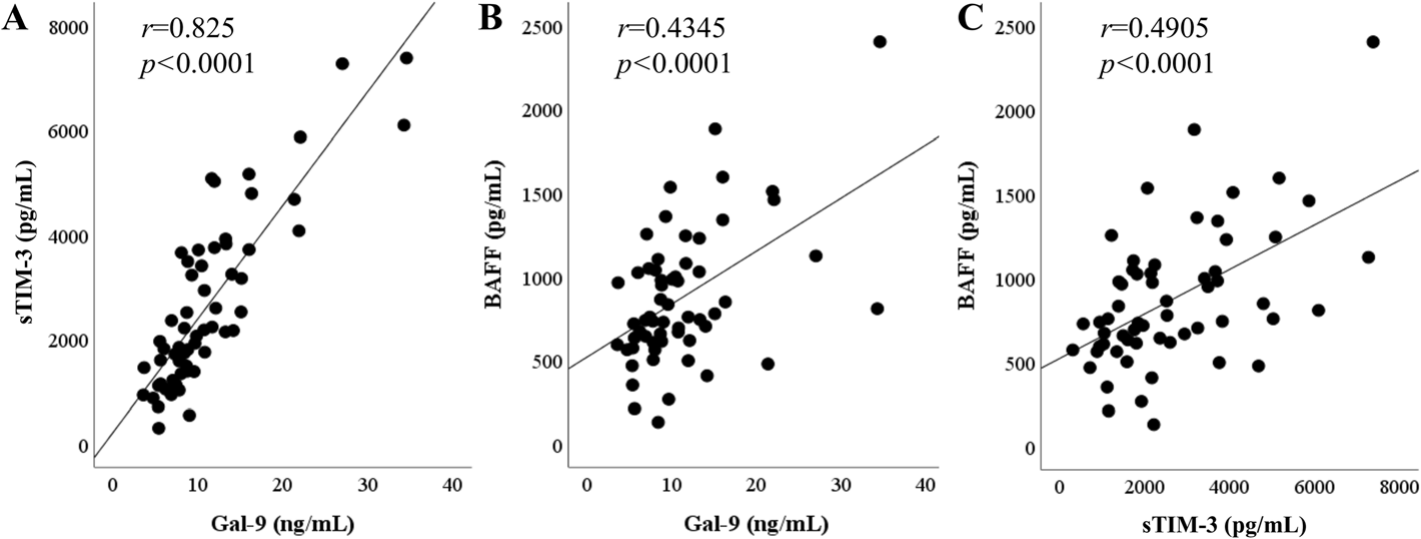
Relationships among the serum sTIM-3, Gal-9, and BAFF in patients with IgG4-RD. **a** Serum Gal-9 was positively correlated with serum sTIM-3. **b**, **c** The serum levels of BAFF were positive in correlation with the serum levels of sTIM-3 and Gal-9. All correlations were determined using Spearman’s rank correlation test. The lines shown are based on simple linear regression

**Fig. 3:**
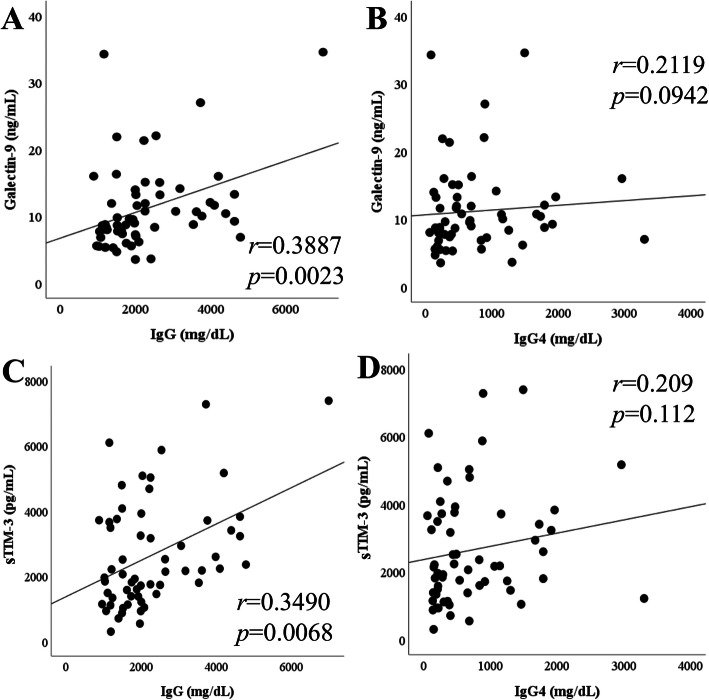
Relationships between the serum levels of checkpoint molecules and IgG subclass in IgG4-RD. **a**, **b** The serum levels of Gal-9 showed a positive correlation with IgG whereas not a significant correlation with IgG4. **c**, **d** The serum levels of sTIM-3 showed a positive correlation with IgG whereas not a significant correlation with IgG4. All correlations were determined using Spearman’s rank correlation test. The lines shown are based on simple linear regression

We performed the correlation analysis among several laboratory markers, including checkpoint molecules and BAFF in IgG4-RD patients. Since BAFF has been reported to be a biomarker for IgG4-RD [18], we measured BAFF as well as checkpoint molecules. Serum levels of Gal-9 or sTIM-3 were positively correlated with the serum levels of (BAFF) (Fig. 2). In addition, the serum levels of Gal-9 or sTIM-3 were positively correlated with serum IgG level; however, there was no significant correlation between the serum levels of these checkpoint molecules and those of IgG4 or the ratio of serum IgG4/IgG (Fig. 3).

### Associations of the serum levels of Gal-9 or sTIM-3 with organ involvement

**Fig. 4:**
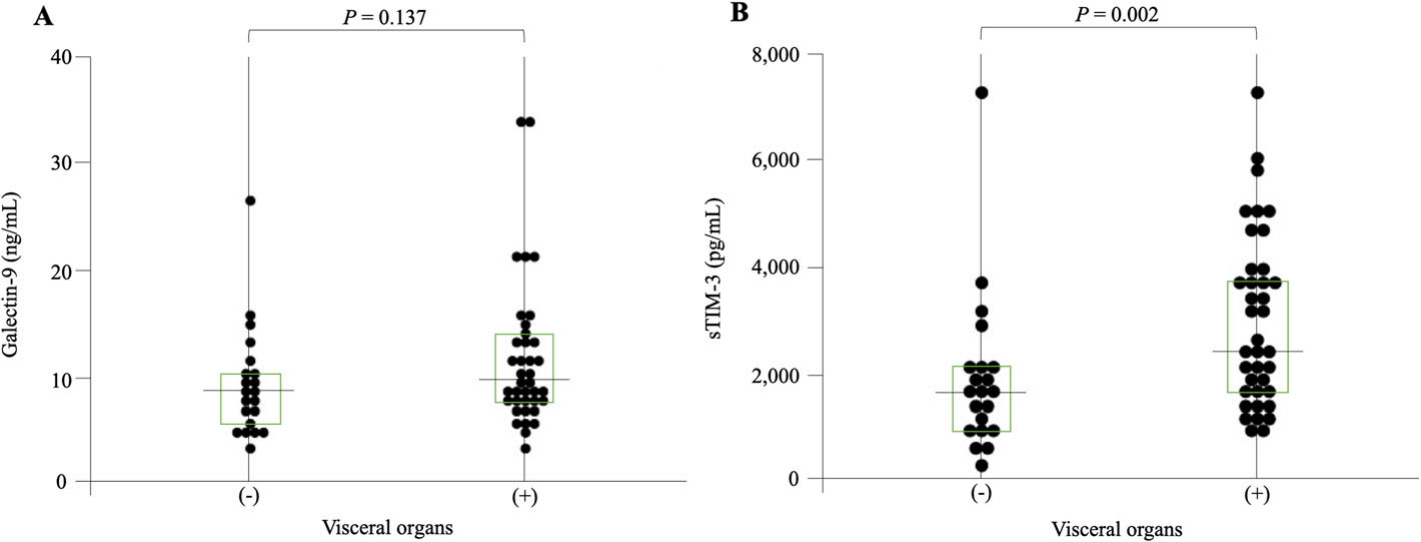
Serum levels of Gal-9 and sTIM-3 with or without visceral organ involvement in IgG4-RD. **a** Serum levels of Gal-9 were not significantly different in the presence of visceral organ involvement. **b** Serum levels of sTIM-3 were significantly higher in IgG4-RD patients with visceral organ lesions compared to those without visceral organ lesions. Statistical significance was determined by the Mann-Whitney *U* test

**Fig. 5:**
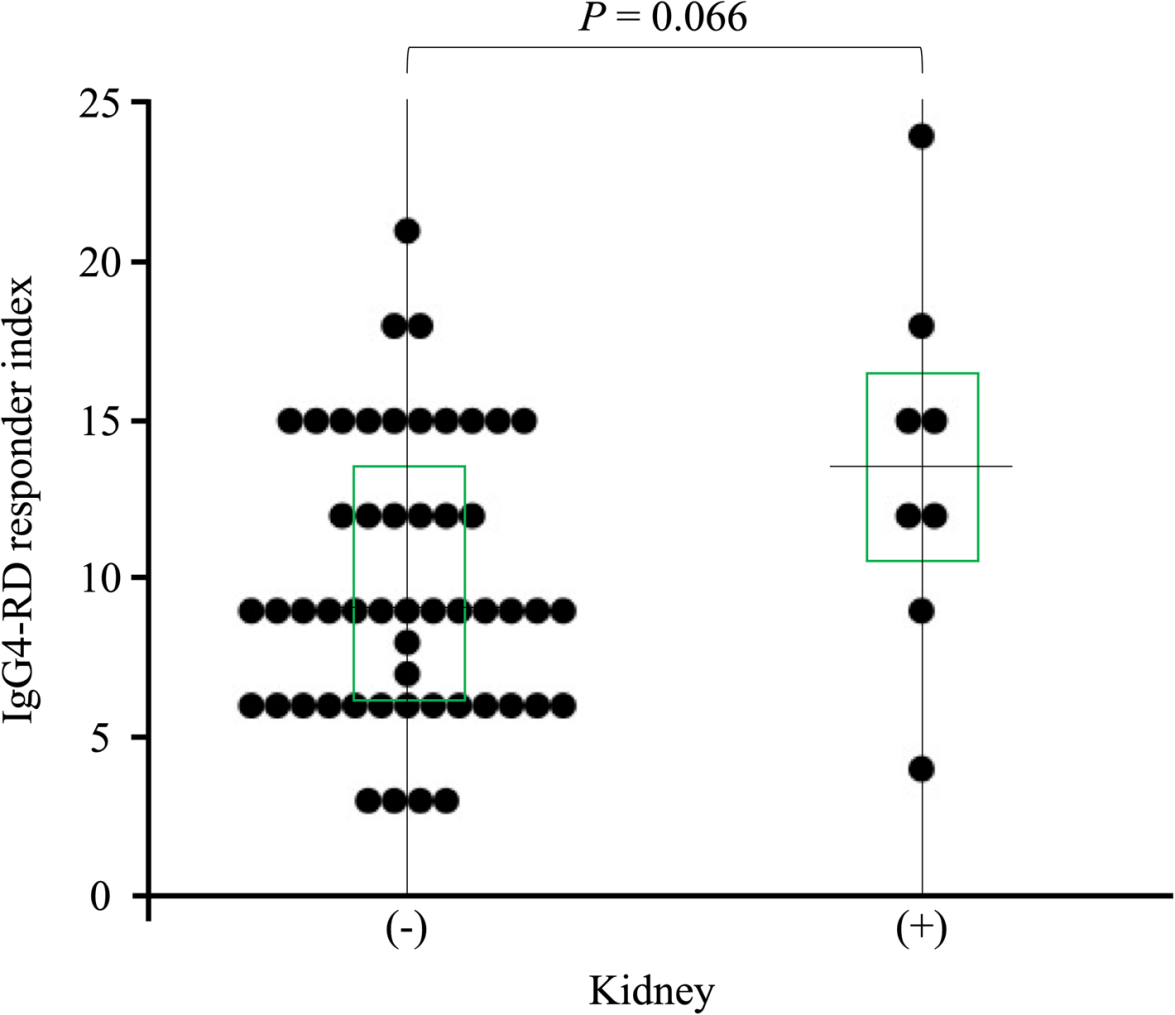
Relationship between IgG4-RD responder index and kidney involvement. IgG4-responder index of IgG4-RD patients with kidney involvement was not significantly higher than that of IgG4-RD patients without kidney involvement. Statistical significance was determined by the Mann-Whitney *U* test

In order to determine whether these checkpoint molecules can be used to differentiate among the IgG4-RD phenotypes, we examined the associations between the serum levels of Gal-9 or sTIM-3 and organ involvements in IgG4-RD patients. All patients with IgG4-RD were subdivided as per the presence of visceral involvements; further, the serum levels of Gal-9 or sTIM-3 were compared between the groups (Fig. 4). There was no significant difference in the serum levels of Gal-9 between IgG4-RD patients with and without the involvement of visceral organs. However, the serum levels of sTIM-3 were significantly higher in patients with visceral involvement than in those without the involvement of visceral organs. We attempted to identify the clinical parameters associations with high sTIM-3 levels (higher than the first quartile of the circulating sTIM-3 level of IgG4-RD; 3656 pg/mL) by performing a multivariate logistic regression analysis (Table 2). The presence of biliary tract (OR, 12.29; 95% confidence interval [95%CI], 1.83–109; *p* = 0.004), kidney (OR, 18.59; 95%CI, 2.4–143; *p* = 0.022), and retroperitoneum (OR, 13.14; 95%CI, 2.3–75.2, *p* = 0.027) involvement was independently associated with high sTIM-3 levels in patients with IgG4-RD. However, the presence of kidney involvement did not affect IgG4-RD RI (Fig. 5).

**Table 2:**
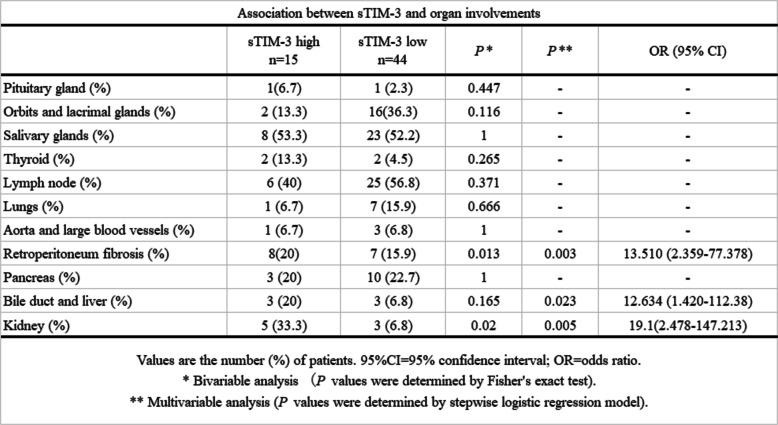
Association between sTIM-3 and organ involvements

### Longitudinal changes in the serum levels of Gal-9 or sTIM-3 after steroid treatment

**Fig. 6:**
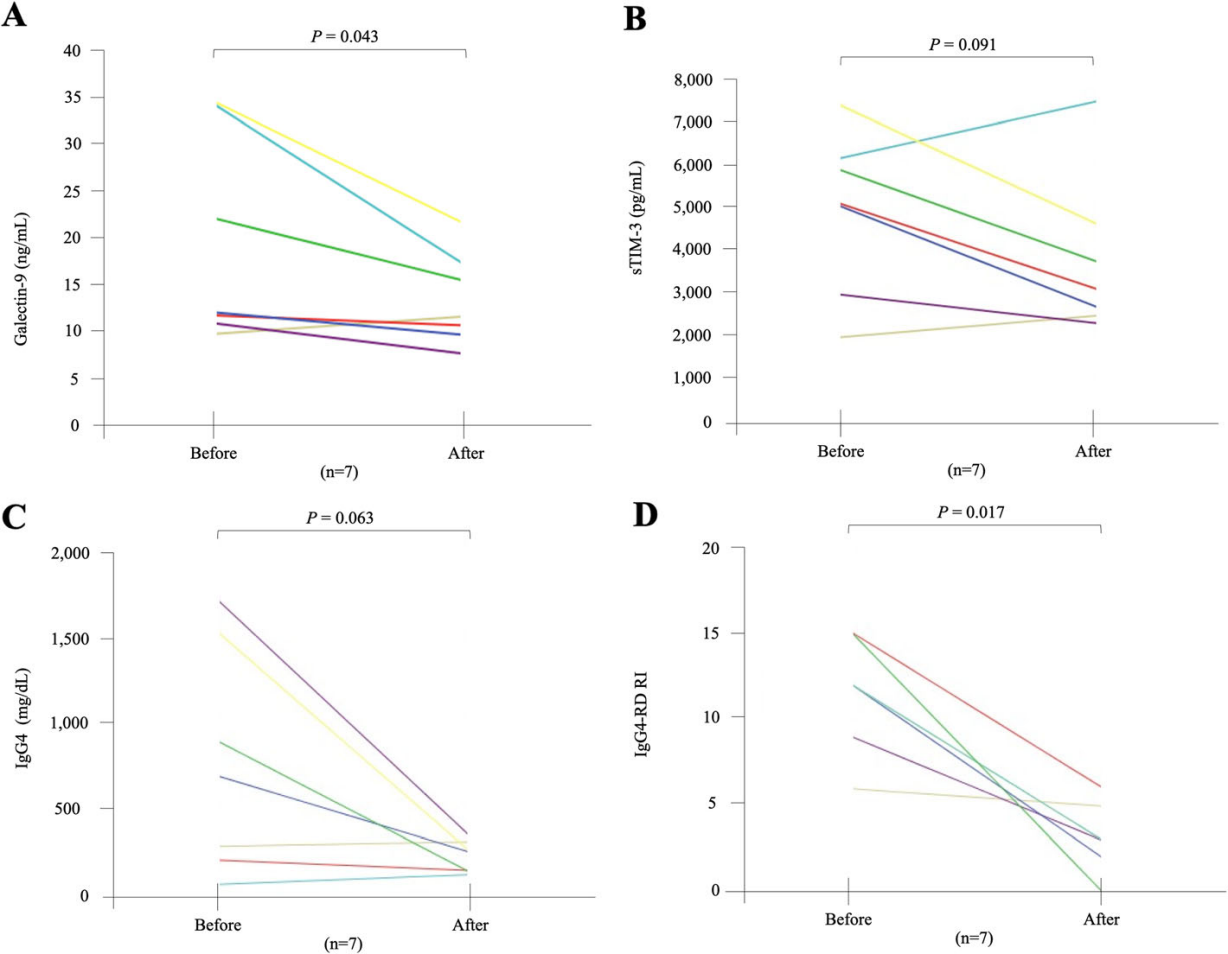
Longitudinal changes of serum Gal-9 or sTIM-3 concentrations in 7 patients with IgG4-RD before and after glucocorticoid therapy. **a**–**c** Gal-9, sTIM-3, and IgG4 concentrations in IgG4-RD patients were not significantly different between before and after treatment. **d** IgG4 responder index was significantly decreased after glucocorticoid therapy. Paired samples from the same subjects were compared by the Wilcoxon signed-rank test

The changes in the serum levels of checkpoint molecules after the induction of steroid treatment in 7 patients are shown in Fig. 6. The median range dose of prednisolone was 35 (25–40) mg/day, and the median duration of steroid treatment was 12 (8.5–49) months. Gal-9 as well as IgG4-RD responder index [19] were reduced in all patients. Although the serum sTIM-3 and serum IgG4 levels tended to decline in many cases, there were no significant differences due to part to a small number of cases.

## Discussion

Galectins, a protein from a family of lectins with affinity for β-galactoside-containing oligosaccharides, are expressed by the immune cells [20]. Recent studies have shown that galectins play crucial regulatory roles in inflammation and autoimmunity [21]. In this study, we evaluated the serum levels of sTIM-3 and its ligand molecule, Gal-9, in IgG4-RD patients. Although circulating Gal-9 or sTIM-3 were not correlated with the serum IgG4 levels or the ratio of IgG4/IgG, our results indicated that the serum levels of Gal-9 and sTIM-3 are significantly elevated in patients with IgG4-RD as compared to those in HCs. These findings indicate that these checkpoint molecules could be involved in the pathophysiology of IgG4-RD.

Previous studies have shown the upregulations of Th2 cytokines (interleukin (IL)-4, IL-5, IL-13, and IL-21) and the regulatory T cell-mediated cytokines (IL-10 and transforming growth factor-β) in IgG4-RD patients [22]. Therefore, IgG4-RD appears to be driven by pathogenic Th2 cells or a combination of Th2 cells and regulatory T cells (Treg cells), and these T cell subsets may activate macrophages and fibroblasts that cause inflammatory and fibrotic processes in the affected organs [23]. We hypothesized that Th1/Th2 imbalance in IgG4-RD is associated with the dysregulation of checkpoint molecules and analyzed their associations with the clinical phenotypes of IgG4-RD. In contrast to patients with organ involvement limited to the lacrimal or salivary glands, patients with visceral involvements presented with higher levels of serum sTIM-3. Furthermore, we observe an organ-specific increment in sTIM-3, particularly biliary, kidney, or retroperitoneum involvement in IgG4-RD. Our data indicated that elevated levels of sTIM-3 in patients with IgG4-RD could be related to the clinical phenotypes of IgG4-RD, including its patterns of organ involvements.

TIM-3 acts as a co-inhibitory receptor that is expressed on exhausted T cells. TIM-3 was initially thought to be expressed only by T cells; it has now been proven that TIM-3 is expressed by multiple cell types, including DCs, macrophages, and Tregs [24], indicating that TIM-3 also functions as an inhibitory receptor in these cells [25]. Gal-9 has been identified as a ligand for TIM-3 [26]; however, its putative ligands other than Gal-9 and its inhibitory effect on T cell remain unclear. The precise cellular interaction within sTIM-3 and its ligands remains unclear; however, it is possible that a combination of sTIM-3 with its ligands competitively reduces the inhibitory effect on the pathway following TIM-3 on immune cells [27], resulting in Th1/Th2 cell imbalance in IgG4-RD [28].

Tfh is a critical regulator of immune responses in inflammatory disorders [29–31]. Checkpoint inhibitors are known to induce B cell activation and class-switched IgG production; these processes are dependent on the Tfh cell function [16]. Furthermore, the case of an IgG4-RD patient with lung cancer receiving checkpoint inhibitors has been reported [14]. These findings suggest that the interaction between B cells and Tfh cells through these checkpoint molecules could be involved in IgG4 class switching [14]. Tfh could be subdivided into distinctive functional subsets as per the checkpoint molecule expressions. Programmed cell death 1 demarcated potent Tfh subsets [32], and TIM-3 appears to be associated with reduced Tfh function [33], suggesting that the co-inhibitory Gal-9/TIM-3 pathway can limit the functions of Tfh [34]. Potent Tfh might play a crucial role in IgG4 class switching [35]; therefore, the elevated levels of sTIM-3 may affect the Tfh functions by interfering with the Gal-9/TIM-3 co-inhibitory checkpoint systems. These co-inhibitory checkpoint pathways may alter the interaction between Tfh cells and plasmablasts [36] in the immunopathology of IgG4-RD and its expanding organ involvement. Given the multi-organ nature of IgG4-RD, the predictors for particular organ involvements would be valuable for clinical application and research of IgG4-RD.

Although the serum concentrations of sTIM-3 were associated with visceral involvements of IgG4-RD, the levels were not modulated by steroid treatment. Steroid is effective for most patients with IgG4-RD [2]; however, the relapses of IgG4-RD were frequently observed during the steroid tapering periods [2]. Steroid is considered to be the first-line treatment for remission induction in IgG4-RD [37]; however, it is possible that the incomplete regulation of immunopathology of IgG4-RD could be linked to this increased serum levels of immuno-checkpoint molecules even after the steroid treatment.

Additional studies are required to clarify the roles of circulating checkpoint molecules in the pathophysiology of IgG4-RD and their relationship to the clinical phenotypes. Furthermore, it is necessary to elucidate whether more aggressive treatment such as B cell depletion treatment may affect the upregulated immune checkpoint molecules in IgG4-RD.

There are certain limitations of this study. The sample size was relatively small, and larger scale studies are necessary to confirm the present finding. Checkpoint molecule profiles in other potential controls, such as those with Sjögren’s syndrome or lymphoproliferative disorders, were not compared with the profiles in IgG4-RD patients in the present study. This study was a cross-sectional analysis for untreated IgG4-RD patients. Therefore, clinical manifestations that occurred during the disease course were not completely surveyed. We enrolled only Japanese patients with IgG4-RD; therefore, non-Japanese patients with IgG4 were not included, and further studies on non-Japanese patients with IgG4-RD are warranted. The mechanism through which the Gal-9/TIM-3 pathway contributes to the pathogenesis of IgG4 was not clarified because the functional analysis by in vitro cannot be performed. However, TIM-3 biology is complex in terms of broad expression by different types of immune cells and multiple ligands. Given the variety of TIM-3 ligands, it is possible that binding to other ligands might facilitate the functions of TIM-3. Moreover, it will be necessary to address whether sTIM-3 acts as an agonist or an antagonist to understand the role of sTIM-3 in IgG4-RD.

## Conclusions

We demonstrated that serum sTIM-3 and Gal-9 levels were elevated in IgG4-RD patients. With respect to the relationship with the clinical phenotypes, the serum sTIM-3 levels were significantly higher in patients with visceral involvements than in those with disease limited to the lacrimal or salivary glands. These data suggest that the circulating checkpoint molecules are involved in the pathophysiology of IgG4-RD and their relationship to its patterns of organ involvement.

## Data Availability

The datasets used and/or analyzed during the current study are available from the corresponding author on reasonable request.

## Abbreviations

IgG4-RD: Immunoglobulin G4-related disease
Treg: Regulatory T cell
Gal-9: Galectin-9
TIM-3: T cell immunoglobulin and mucin-containing-molecule-3
sTIM-3: Soluble T cell immunoglobulin and mucin-containing-molecule-3
RA: Rheumatoid arthritis
IL: Interleukin
Tfh: T follicular helper T cell
Th1: Type 1 helper T cell
Th2: Type 2 helper T cell
